# Transcriptome Analysis Reveals Distinct Differences in Organic Acid Metabolism Between the Pericarp and the Pulp of *Cerasus humilis* During Fruit Maturation

**DOI:** 10.3390/plants14071105

**Published:** 2025-04-02

**Authors:** Bingcheng Guo, Li Zhang, Jinli Guo

**Affiliations:** College of Horticulture and Plant Protection, Inner Mongolia Agricultural University, Hohhot 010010, China; guobingcheng111@163.com (B.G.); zlkx628@163.com (L.Z.)

**Keywords:** *Cerasus humilis*, enzyme activity, gene expression organic acid metabolism, transcriptomic analysis

## Abstract

Organic acids are key components that determine the taste and flavor of fruits, playing a crucial role in maintaining fruit quality and nutritional value. To investigate the metabolic differences of organic acids between the fruit pericarp and the pulp during the developmental maturation of the *Cerasus humilis*, this experiment utilized *Cerasus humilis* cultivated in Inner Mongolia, China, as the experimental material. By measuring the malic acid and citric acid content, as well as the activities of the related metabolic enzymes in the fruit pericarp and the pulp at five developmental stages, this study investigated the characteristics of organic acid accumulation, changes in enzyme activities, and the expression trends of corresponding genes. Transcriptomic data were integrated to support the analysis. This study specifically analyzed the reasons for the differences in acidity between the pericarp and the pulp, and performed a correlation analysis of various indicators. The results indicated that, during development, the organic acid composition in both the pericarp and the pulp was primarily malic acid, with citric acid as a secondary component. The malic acid and citric acid content in the pericarp were significantly higher than in the pulp, resulting in greater overall acidity in the pericarp. The combined action of PEPC, NAD-MDH, and NADP-ME was identified as the primary reason for the differences in malic acid content between the pericarp and the pulp of *Cerasus humilis*. CS and ACO were identified as the key enzymes responsible for the lower citric acid content in the pulp compared to the pericarp. Furthermore, the expression levels of *ChMDH2*, *ChME*, *ChCS2*, *ChCS3*, *ChACO1*, and *ChACO2* differed significantly between the fruit pericarp and the pulp, suggesting their regulatory roles in organic acid accumulation.

## 1. Introduction

*Cerasus humilis* (Bge.) Sok. is a member of the subgenus *Microcerasus* in the *Rosaceae* family. It is a unique wild fruit tree resource in China, widely distributed in regions such as Shanxi, Inner Mongolia, Hebei, and Heilongjiang [1]. The fruit of *Cerasus humilis* has a distinctive flavor and is rich in various amino acids, vitamins, and minerals. The fruit is characterized by its high acidity, with the titratable acid content ranging from 1.0% to 2.0%, which is higher than that found in most other drupe fruits. The leaves of *Cerasus humilis* can be used to make tea, and its kernels have medicinal applications. The plant holds significant nutritional, economic, and medicinal value [2], offering broad prospects for further development [3]. The *Cerasus humilis* industry has long faced challenges related to the fruit’s palatability, primarily due to its high organic acid content. This imbalance in acid metabolism has hindered the development of a fresh food market, leading to a focus on processing *Cerasus humilis* fruit into juices and wines, and contributing to the homogenization of the processing industry.

Organic acids are key nutritional components of fruits, significantly affecting their flavor. In plant physiology, they are involved in numerous metabolic pathways during fruit development, including respiration and photosynthesis, and play a crucial role in the synthesis of esters, phenolics, and amino acids [4]. Organic acids are generated through metabolic processes involving pyruvate and are degraded via gluconeogenesis. The tricarboxylic acid cycle (TCA cycle) facilitates the interconversion of organic acid components, which then undergo additional metabolic reactions [5,6].

The composition and content of organic acids vary significantly across different tree species and at various stages of fruit development. Fruits are typically classified into three categories—malic, citric, and tartaric acid fruits—based on the predominant organic acids. The acidity of these acids differs, but they all initially increase and then decrease during fruit development and maturation. Furthermore, the composition and content of organic acids also vary across different parts of the fruit [7]. In studies on kumquats [8], grapes [9], and grapefruit [10], significant differences were observed in the composition and content of organic acids between the pericarp and the pulp.

The organic acid metabolic process has been well studied, and the regulation of organic acid metabolism in fruits is significantly complex. This metabolic process is not independently regulated by a single enzyme but relies on a synergistic network of multiple enzyme systems [11,12]. There are significant differences in the key enzymes and their enzymatic functions in the organic acid metabolic pathways between different fruit types. For example, malate dehydrogenase (MDH) plays a central role in the TCA cycle of apple fruit [13], while citrate synthase (CS) dominates citric acid accumulation in citrus fruits [14,15].

*Cerasus humilis* is a fruit tree known for its significant acidity, exhibiting particularly high acidity during its immature stage. It serves as a model for studying organic acid metabolism due to the marked changes in organic acid concentrations throughout its development. In mature fruit, the predominant organic acid is malic acid, with citric acid present in smaller amounts. Studying the accumulation patterns of organic acids in *Cerasus humilis* and the trends in the related metabolic enzyme activities provides a theoretical basis for exploring the nutritional quality of this species. Current research on *Cerasus humilis* is extensive, with reports documenting changes in enzyme activities associated with organic acid metabolism in the fruit, and it focuses on organic acids and their associated metabolic enzyme activities [16]. However, the differences in the organic acid metabolism between the pericarp and the pulp of *Cerasus humilis* have not been previously reported. This study uses *Cerasus humilis* as materials and analyzes the physiomics data in conjunction with the transcriptome data to systematically compare the composition and content of organic acids, the activities of acid-metabolizing enzymes, and changes in gene expression between the pericarp and the pulp. The relationships among these factors are discussed, which will help deepen the understanding of organic acid metabolism in *Cerasus humilis* and provide a theoretical basis for further research on organic acids and subsequent regulation.

## 2. Results

### 2.1. Analysis of Organic Acid Components and Content

**Figure 1:**
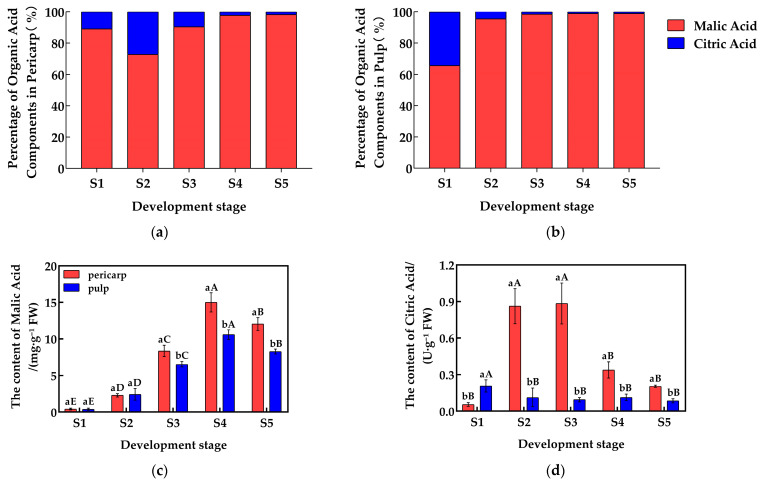
Changes in the proportions and content of organic acid components in the pericarp and the pulp of *Cerasus humilis* during development: (**a**) represents the proportion of acid content in the pericarp of the *Cerasus humilis*; (**b**) represents the proportion of acid content in the pulp of the *Cerasus humilis*; (**c**) represents the changes in malic acid content in both the pericarp and the pulp of the *Cerasus humilis* during development; (**d**) represents the changes in citric acid content in both the pericarp and the pulp of the *Cerasus humilis* during development. S1: the young fruit stage; S2: the hard seed stage; S3: the coloring–enlargement stage; S4: the hard–ripe stage; S5: the fully ripe stage. Note: lowercase letters represent the results of Duncan’s test at *p* < 0.05 for the same developmental stage of the pericarp and the pulp, while uppercase letters represent the results of Duncan’s test at *p* < 0.05 for the pericarp and the pulp at different developmental stages, respectively; the error bars represent the average standard deviation (n = 3).

The malic acid (Mal) and citric acid (CA) contents in the pericarp and the pulp of *Cerasus humilis* during development were quantitatively analyzed, and the proportions of each organic acid component are shown in Figure 1a,b. The malic acid content in both the pericarp and the pulp initially increased and then decreased, reaching their highest levels at the fully ripe stage, with proportions of 98.66% and 98.88%, respectively. The citric acid content in the pericarp followed a similar trend, peaking at the hard seed stage with a proportion of 27.24%; while, in the pulp, citric acid content gradually decreased, reaching the lowest percentage (<2%) at the fully ripe stage. Malic acid dominates (>95%), with citric acid as a minor component. From the above, it can be seen that the proportion of citric acid generally decreased throughout development, while the proportion of malic acid increased, making malic acid the primary acid component in both the pericarp and the pulp.

A detailed analysis was performed on the changes in the content of common organic acids in the pericarp and the pulp of *Cerasus humilis* at different developmental stages, with the results presented in Figure 1. The malic acid content in both the pericarp and the pulp initially increased and then decreased as the fruit developed, reaching peak values of 15.01 mg/g in the pericarp and 10.59 mg/g in the pulp during the hard–ripe stage. Malic acid content in the pericarp accumulated rapidly after the hard seed stage, and during the coloring–enlargement, the hard–ripe, and the fully ripe stages, the pericarp content was significantly higher than that in the pulp (Figure 1c). Similarly, the citric acid content in the pericarp followed an increasing then decreasing trend, peaking at 0.88 mg/g during the coloring–enlargement stage. However, the citric acid content in the pulp did not show a clear trend. Citric acid in the pericarp accumulated rapidly after the young fruit stage, with significantly higher levels in the pericarp compared to the pulp during the hard seed stage, the coloring–enlargement stage, the hard–ripe stage, and the fully ripe stage (Figure 1d). These results highlight the distinct accumulation patterns of malic acid and citric acid between the pericarp and the pulp.

### 2.2. Changes in the Activity of Enzymes Related to Acid Metabolism

The organic acid metabolic process is controlled by multiple enzymes. Phosphoenolpyruvate (PEP) is converted into oxaloacetic acid (OAA) by the catalysis of phosphoenolpyruvate carboxylase (PEPC). OAA can then be converted into malic acid through the catalysis of NAD-malic dehydrogenase (NAD-MDH). The generated malic acid will also be decomposed by NADP-malic enzyme (NADP-ME). Citric acid, catalyzed by citrate synthase (CS), is formed by the condensation of acetyl-CoA and oxaloacetate, and it is further degraded to α-ketoglutarate by aconitase.

**Figure 2:**
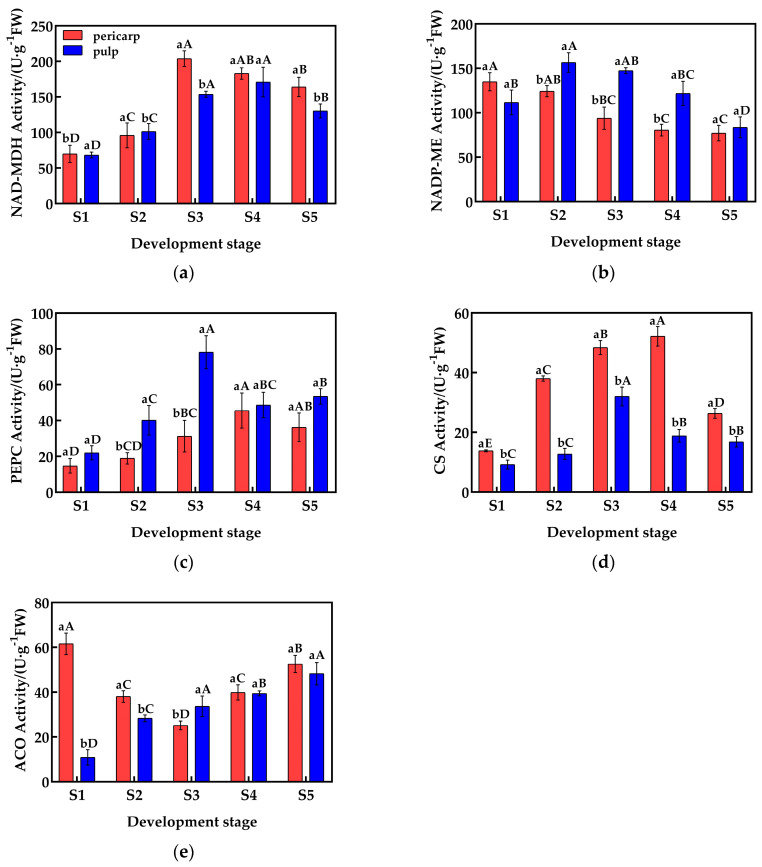
Changes in the activity of enzymes related to acid metabolism in the pericarp and the pulp of *Cerasus humilis*: (**a**) represents changes in NAD-MDH activity during development; (**b**) represents changes in NADP-ME activity during development; (**c**) represents changes in PEPC activity during development; (**d**) represents changes in CS activity during development; (**e**) represents changes in ACO activity during development. S1: the young fruit stage; S2: the hard seed stage; S3: the coloring–enlargement stage; S4: the hard–ripe stage; S5: the fully ripe stage. Note: lowercase letters represent the results of Duncan’s test at *p* < 0.05 for the same developmental stage of the pericarp and the pulp, while uppercase letters represent the results of Duncan’s test at *p* < 0.05 for the pericarp and the pulp at different developmental stages, respectively; the error bars represent the average standard deviation (n = 3).

During fruit development, the activity of malate dehydrogenase (NAD-MDH) in the pericarp and the pulp of *Cerasus humilis* is shown in Figure 2a. The activity of NAD-MDH in both the pericarp and the pulp is similar, with a trend of first increasing and then decreasing, and the highest activity was observed during the coloring–enlargement stage (203.90 U·g^−1^) and the hard–ripe stage (170.87 U·g^−1^). There was no significant difference in NAD-MDH activity between the pericarp and the pulp during the young fruit stage, the hard seed stage, and the hard–ripe stage; but, in the coloring–enlargement stage and the fully ripe stage, NAD-MDH activity in the pericarp was significantly higher than in the pulp.

The activity of NADP-ME in the pericarp and the pulp exhibited different trends and magnitudes (Figure 2b). In the pericarp, NADP-ME activity exhibited a gradual decrease, reaching the lowest value at maturity with an activity of 77.11 U·g^−1^. By contrast, the activity in the pulp first increased and then decreased, with the highest activity at the hard-core stage (156.50 U·g^−1^). NADP-ME activity in the pulp was significantly higher than in the pericarp during the hard seed stage, the coloring–enlargement stage, and the hard–ripening stage, while there were no significant differences during the young fruit and fully ripe stages.

The activity of PEPC in the pericarp and the pulp differed in magnitude, but the trends were similar (Figure 2c). Both the pericarp and the pulp exhibited an increasing followed by a decreasing trend in PEPC activity, with the highest activity observed at the hard–ripe stage and the coloring–enlargement stage (78.15 U·g^−1^ and 45.58 U·g^−1^, respectively). In the hard seed stage, the coloring–enlargement stage, and the fully ripe stage, PEPC activity in the pulp was significantly higher than in the pericarp, while no significant differences were observed in the young fruit stage and the hard–ripe stage.

The activity of CS in the pericarp and the pulp varied in magnitude, but the trends were similar (Figure 2d). Both the pericarp and the pulp exhibited an increasing followed by a decreasing trend in CS activity, with the highest activity observed at the hard–ripe stage and the coloring–enlargement stage (55.46 U·g^−1^ and 32.01 U·g^−1^, respectively). From the young fruit stage to the fully ripe stage, CS activity in the pericarp was significantly higher than in the pulp, with activity differences nearing a threefold difference during the hard seed stage and the hard–ripe stage.

The activity of ACO in the pericarp and the pulp exhibited different trends and magnitudes (Figure 2e). In the pericarp, ACO activity first decreased and then increased, reaching the lowest value at the coloring–enlargement stage (25.12 U·g^−1^), while in the pulp, ACO activity gradually increased, reaching the highest value at the fully ripe stage (48.22 U·g^−1^). During the young fruit and hard seed stages, ACO activity in the pericarp was significantly higher than in the pulp, while at the coloring–enlargement stage, ACO activity in the pulp was significantly higher than in the pericarp. At the hard–ripe stage and the fully ripe stage, no significant differences were observed.

In summary, the activity of enzymes related to malic acid and citric acid metabolism in the pericarp and the pulp exhibited varying degrees of differences, which may be the main reason for the differences in organic acid content.

### 2.3. Screening of Genes Involved in Organic Acid Metabolism Differences Between the Pericarp and the Pulp of Cerasus humilis During Development

#### 2.3.1. Differential Gene Analysis of the Transcriptome Between the Pericarp and the Pulp

Differential expression analysis was performed between the pericarp and the pulp at the same developmental stages: S3GP_vs_S3GR and S4GR_vs_S4GP, with the screening criteria set as fold change ≥ 2 and FDR ≤ 0.01.

**Figure 3:**
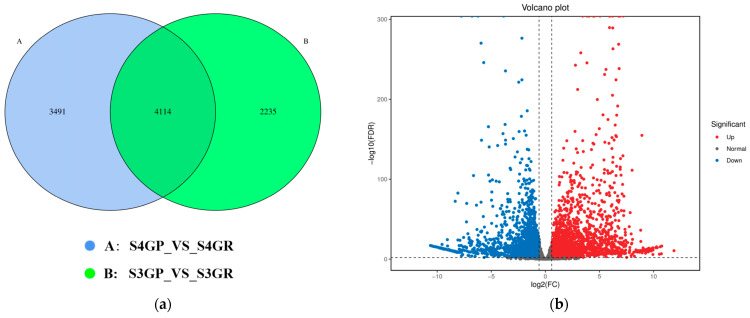

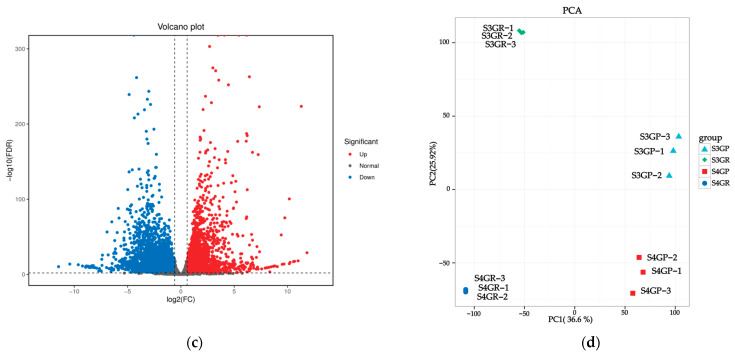
Differentially expressed genes between the pericarp and the pulp of *Cerasus humilis* during the coloring–enlargement stage and the hard–ripe stage: (**a**) represents the Venn diagram of differentially expressed genes in the pericarp and the pulp during the coloring–enlargement stage and the hard–ripe stage; (**b**) represents the volcano plot of differentially expressed genes in the pericarp and the pulp during the coloring–enlargement stage; (**c**) represents the volcano plot of differentially expressed genes in the pericarp and the pulp during the hard–ripe stage. The x-axis represents a log2 fold change, and the y-axis represents a negative log10 *p*-value. Significantly upregulated genes are in red, downregulated genes in blue, and nonsignificant changes in gray; (**d**) represents the principal component analysis (PCA) plots of pericarp and pulp. S3: the coloring–enlargement stage; S4: the hard–ripe stage; GP: the pericarp of *Cerasus humilis*; GR: the pulp of *Cerasus humilis*. Note: Each point in the volcano plot represents a gene, with the x-axis showing the logarithmic value of the fold change in gene expression between the two samples, and the y-axis showing the negative logarithmic value of the statistical significance of gene expression changes. In the plot, blue points represent downregulated differentially expressed genes, red points represent upregulated differentially expressed genes, and gray points represent non-differentially expressed genes.

There are 3491 unique differentially expressed genes in the pericarp and the pulp during the S3 stage, 2235 unique differentially expressed genes in the pericarp and the pulp during the S4 stage, and 4114 common differentially expressed genes between the two stages (Figure 3a). The results exhibited that there were a total of 7605 differentially expressed genes between the pericarp and the pulp during the S3 stage, with 3471 upregulated genes and 4134 downregulated genes (Figure 3b). During the S4 stage, 6349 differentially expressed genes were found, with 3307 upregulated genes and 3042 downregulated genes (Figure 3c).

#### 2.3.2. Differential Gene Enrichment Analysis

Perform a functional annotation of differentially expressed genes in the database and statistical analysis of the number of genes annotated in each differentially expressed gene set. The detailed content is shown in Table 1.

#### 2.3.3. KEGG Annotation and Pathway Enrichment Analysis of Differentially Expressed Genes

**Figure 4:**
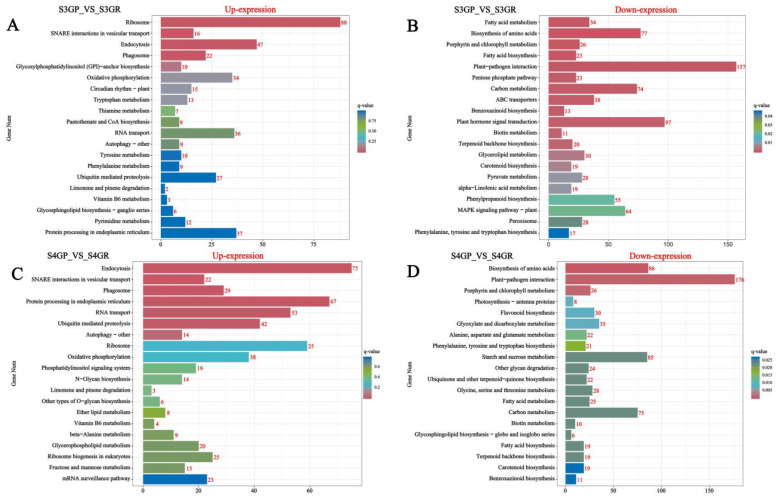
Pathway enrichment analysis of differentially expressed genes in the pericarp and the pulp of *Cerasus humilis* during the coloring–enlargement stage and the hard–ripe stage: The horizontal axis represents GeneNum, the number of genes of interest annotated in the entry, while the vertical axis represents each pathway entry. The colors of the bars indicate the q-value from the hypergeometric test: (**A**) represents S3GP vs. S3GR upregulated; (**B**) represents S3GP vs. S3GR downregulated; (**C**) represents S4GP vs. S4GR upregulated; (**D**) represents S4GP vs. S4GR downregulated.

KEGG annotation and pathway enrichment analysis of differentially expressed genes were performed, and the results are shown in the figure below. The results show that, for S3GP_vs_S3GR, all differentially expressed genes were enriched in 134 pathways according to the annotation results (Figure 4A,B); for S4GP_vs_S4GR, all differentially expressed genes were enriched in 132 pathways according to the annotation results (Figure 4C,D). The genes upregulated in GP relative to GR during the S3 and S4 phases involved pathways such as ribosomal, endocytosis, oxidative phosphorylation, and endoplasmic reticulum protein processing pathways. However, genes downregulated in GP relative to GR at the S3 and S4 phases involved pathways such as plant–pathogen interaction, fatty acid metabolism, fatty acid biosynthesis, and phytohormone signaling.

#### 2.3.4. Prediction of Genes Related to Organic Acid Metabolic Pathways

The predicted results and naming of the unknown genes are shown in the Table 2.

### 2.4. Expression Analysis of Genes Encoding Enzymes Related to Acid Metabolism

An analysis of the organic acid content in both the pericarp and the pulp of *Cerasus humilis* reveals that malic acid is the primary component, with citric acid following. This suggests that the combined levels of malic acid and citric acid account for the acidity variation between the pericarp and the pulp of *Cerasus humilis*. The coloring–enlargement stage and the hard–ripe stage are crucial stages in the development of *Cerasus humilis*, characterized by the most significant changes. Based on the transcriptome sequencing results of the pericarp and the pulp during the coloring–enlargement stage and the hard–ripe stage, and in comparison with homologous sequences from the National Center for Biotechnology Information (NCBI) database (https://www.ncbi.nlm.nih.gov/, accessed on 15 February 2025), changes in malic acid and citric acid metabolism related genes during the growth and development of the pericarp and the pulp of *Cerasus humilis* were analyzed.

**Figure 5:**
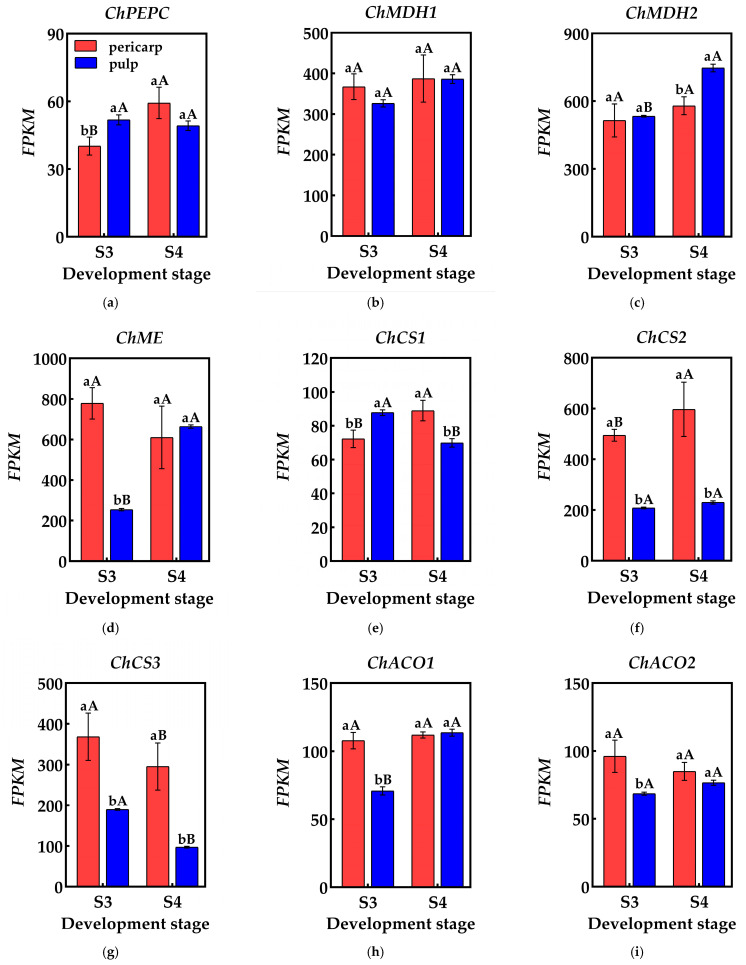
Changes in genes related to organic acid metabolism in the pericarp and the pulp of *Cerasus humilis*: (**a**) represents *PEPC* gene expression; (**b**) represents *ChMDH1* gene expression; (**c**) represents *ChMDH2* gene expression; (**d**) represents *ChME* gene expression; (**e**) represents *ChCS1* gene expression; (**f**) represents *ChCS2* gene expression; (**g**) represents *ChCS3* gene expression; (**h**) represents *ChACO1* gene expression; (**i**) represents *ChACO2* gene expression. S3: the coloring–enlargement stage; S4: the hard–ripe stage. Note: lowercase letters represent the results of Duncan’s test at *p* < 0.05 for the same developmental stage of the pericarp and the pulp, while uppercase letters represent the results of Duncan’s test at *p* < 0.05 for the pericarp and the pulp at different developmental stages, respectively; the error bars represent the average standard deviation (n = 3).

Organic acid metabolism during fruit development is highly dynamic, with fluctuations in components and content driven by a cascade of reactions involving multiple genes and enzymes. Phosphoenolpyruvate carboxylase (PEPC) influences the synthesis of malic acid and citric acid by catalyzing the β-carboxylation of phosphoenolpyruvate (PEP) to produce oxaloacetic acid (OAA) and inorganic phosphate. Researchers have analyzed the transcriptome database of the pericarp and the pulp of *Cerasus humilis* and identified at least one gene from the phosphoenolpyruvate carboxylase (PEPC) family. During the coloring–enlargement stage and the hard–ripe stage, expression levels of *ChPEPC* increases in the pericarp but decreases in the pulp. The expression levels of *ChPEPC* in both tissues remain low, aligning with the observed changes in PEPC activity (Figure 5a).

Malate dehydrogenase (NAD-MDH) aids in malic acid accumulation by catalyzing the conversion of oxaloacetic acid (OAA) to malic acid. A transcriptome analysis identified at least two members of the malate dehydrogenase (NAD-MDH) gene family. During the coloring–enlargement stage and the hard–ripe stage, the expression levels of the *ChMDH1* and *ChMDH2* genes increased in both the pericarp and the pulp. Notably, expression levels of *ChMDH2* in the pulp significantly surpassed that in the pericarp during the hard–ripe stage. The elevated expression levels of the *ChMDH1* and *ChMDH2* genes in both the pericarp and the pulp likely contributes to the increased NAD-MDH activity (Figure 5b,c).

Malic enzyme (NADP-ME) facilitates malic acid breakdown by catalyzing the conversion of malic acid to phosphoenolpyruvate (PEP). Transcriptome data identified at least one gene encoding malic enzyme (NADP-ME). During the coloring–enlargement stage and the hard–ripe stage, expression levels of *ChME* decreases in the pericarp but increases in the pulp, aligning with the changes in NADP-ME activity in both tissues throughout development. During the coloring–enlargement stage, the expression level of *ChME* was significantly higher in the pericarp than in the pulp. However, during the hard–ripe stage, this trend reversed, though not significantly, likely contributing to the higher NADP-ME activity in the pulp (Figure 5d).

Citrate synthase (CS) catalyzes the condensation of acetyl-CoA and oxaloacetic acid (OAA), promoting citric acid accumulation. A transcriptome analysis identified at least three genes encoding citrate synthase (CS). During the coloring–enlargement stage and the hard–ripe stage, the expression levels of the *ChCS2* and *ChCS3* genes were higher than *ChCS1*, with higher expression in the pericarp compared to the pulp. In the pericarp, the expression levels of the *ChCS1* and *ChCS2* genes increase, while *ChCS3* decreases. In the pulp, expression levels of *ChCS1* and *ChCS3* decrease, while *ChCS2* follows an upward–downward pattern. Differential expression of ChCS2 and ChCS3 may be the main reason for the difference in CS activity between the pericarp and the pulp, which in turn affects the citric acid content of both (Figure 5e–g).

Aconitase (ACO), crucial for citric acid degradation in the tricarboxylic acid cycle, is encoded by at least two genes identified in the transcriptome data. During the coloring–enlargement and hard–ripe stages, the expression levels of *ChACO1* and *ChACO2* in the pericarp remain relatively stable, while *ChACO1* expression in the pulp gradually increases. During the coloring–enlargement stage, the expression levels of *ChACO1* and *ChACO2* in the pericarp is higher than in the pulp, with no significant differences observed at the hard–ripe stage. These expression trends in the pulp align with changes in ACO activity, facilitating citric acid degradation (Figure 5h,i).

### 2.5. qRT-PCR Validation

**Figure 6:**
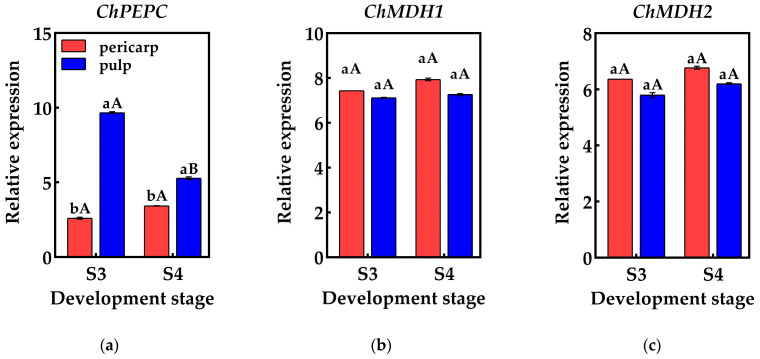

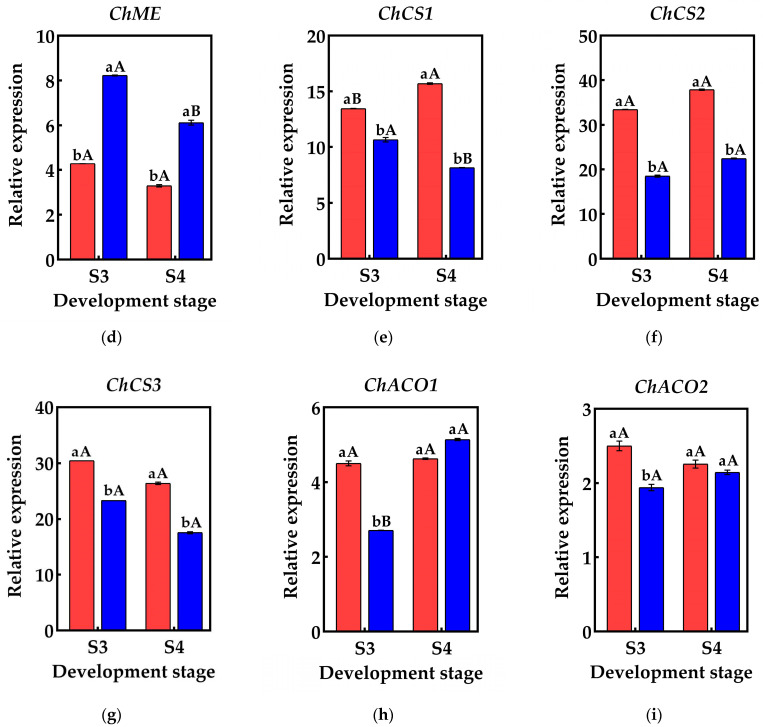
Changes in the relative expression of genes related to organic acid metabolism in the pericarp and the pulp of *Cerasus humilis*: (**a**) represents the relative expression of *PEPC*; (**b**) represents the relative expression of *ChMDH1*; (**c**) represents the relative expression of *ChMDH2*; (**d**) represents the relative expression of *ChME*; (**e**) represents the relative expression of *ChCS1*; (**f**) represents the relative expression of *ChCS2*; (**g**) represents the relative expression of *ChCS2*; (**h**) represents the relative expression of *ChACO1*; (**i**) represents the relative expression of *ChACO2*. S3: the coloring–enlargement stage; S4: the hard–ripe stage. Note: lowercase letters represent the results of Duncan’s test at *p* < 0.05 for the same developmental stage of the pericarp and the pulp, while uppercase letters represent the results of Duncan’s test at *p* < 0.05 for the pericarp and the pulp at different developmental stages, respectively; the error bars represent the average standard deviation (n = 3).

To validate the reliability of the RNA-Seq data, qRT-PCR was employed to detect the genes associated with organic acid metabolism predicted by the screening process. The results indicated that the relative expression of these genes differed between the S3 and S4 pericarp and pulp, with similar gene expression patterns and consistent data trends in both qRT-PCR and RNA-Seq. This suggests that the genes identified in this study, which are involved in the accumulation and metabolism of organic acids in *Cerasus humilis*, are reliable (Figure 6).

### 2.6. Correlation Analysis

#### 2.6.1. The Correlation Between Acid Content in the Pericarp and the Pulp of *Cerasus humilis* and the Activity of Enzymes Involved in Acid Metabolism

**Figure 7:**
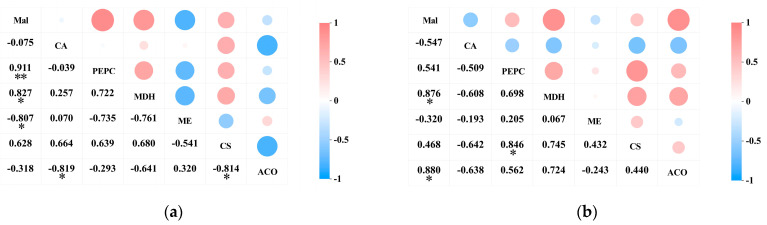
The correlation between acid content and the activity of acid metabolism-related enzymes in the pericarp and the pulp of *Cerasus humilis*: (**a**) represents the relationship between organic acids and their related metabolic enzymes in the pericarp; (**b**) represents the relationship between organic acids and their related metabolic enzymes in the pulp. Note: “*” indicates a significant correlation at the *p* < 0.05 level, and “**” indicates an extremely significant correlation at the *p* < 0.01 level.

A correlation analysis was conducted on the acid content and the activity of acid metabolism-related enzymes in the pericarp and the pulp of *Cerasus humilis* during development. The results indicated a positive correlation between malic acid content and PEPC activity in both the pericarp and the pulp of *Cerasus humilis*, with a highly significant correlation observed in the pericarp (*p* < 0.01). A significant positive correlation was observed between malic acid content and NAD-MDH activity in both the fruit pericarp and the pulp (*p* < 0.05). A negative correlation was found between malic acid content and NADP-ME activity in both the pericarp and the pulp, with a significant correlation in the pericarp (*p* < 0.05) (Figure 7a).

The citric acid content in both the pericarp and the pulp exhibited a negative correlation with PEPC activity, though this correlation was not significant. A positive correlation was observed between citric acid content in the pericarp and CS activity, whereas a negative correlation was found between citric acid content in the pulp and CS activity; however, neither correlation was significant. A negative correlation was observed between citric acid content in both the pericarp and the pulp and ACO activity, with a significant correlation in the pericarp (*p* < 0.05) (Figure 7b).

These results indicate that PEPC and NAD-MDH facilitate the synthesis of malic acid, while NADP-ME aids its degradation; CS promotes citric acid synthesis, and ACO promotes its breakdown. However, the acid metabolism mechanisms in the pericarp and the pulp may vary, with the two organic acids showing different correlations with the activity of their respective metabolic enzymes. They interact synergistically in the metabolic processes of malic and citric acid.

#### 2.6.2. Relationship Between the Activity of Acid Metabolism-Related Enzymes and the Expression of Their Encoding Genes in the Pericarp and the Pulp of *Cerasus humilis*

**Figure 8:**
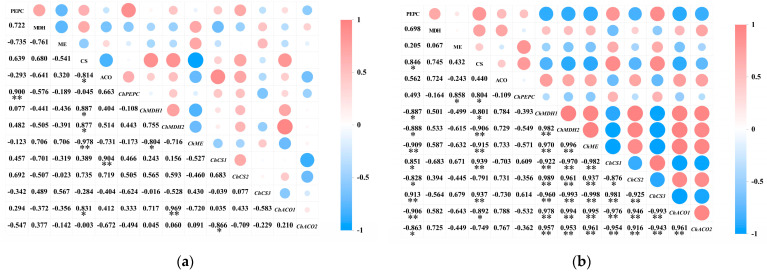
The relationship between the activity of acid metabolism-related enzymes and the expression of their encoding genes in the pericarp and the pulp of *Cerasus humilis*: (**a**) represents the relationship between acid content and enzyme activity in the pericarp and the pulp of *Cerasus humilis* during development; (**b**) represents the relationship between acid metabolism enzyme activity and their encoding genes in the pulp of *Cerasus humilis*. Note: “*” indicates a significant correlation at the *p* < 0.05 level, and “**” indicates an extremely significant correlation at the *p* < 0.01 level.

A correlation analysis was performed separately on the activity of acid metabolism-related enzymes and their encoding genes in the pericarp and the pulp of *Cerasus humilis* during development (Figure 8). The results indicated that PEPC activity in both the pericarp and the pulp was positively correlated with *ChPEPC* expression, with a highly significant correlation in the pericarp (*p* < 0.01). MDH activity in both the pericarp and the pulp was positively correlated with *ChMDH1* and *ChMDH2* expression to varying degrees, with a stronger correlation in the pulp than in the pericarp. ME activity in the pericarp was positively correlated with *ChME* expression; whereas, in the pulp, it was negatively correlated, although neither correlation was significant. CS activity in the pericarp was positively correlated with *ChCS1* and *ChCS2* expression to varying extents; while, in the pulp, it was negatively correlated with *ChCS1* and *ChCS2* expression, with a highly significant correlation with *ChCS1* (*p* < 0.01). ACO activity in the pericarp was positively correlated with *ChACO1* and negatively correlated with *ChACO2* expression; whereas, in the pulp, both were positively correlated, but none of these correlations reached significance.

These results suggest that the same enzyme and gene exhibit differential expression between the pericarp and the pulp, with varying degrees of correlation between the activity of acid metabolism-related enzymes and their encoding genes.

## 3. Discussion

### 3.1. Changes in Organic Acid Content in the Pericarp and the Pulp of Cerasus humilis During Development

During the development and maturation of *Cerasus humilis*, malic acid and citric acid are the primary organic acids contributing to its acidity [6,17]. Malic acid provides a sharp, immediate sourness, while citric acid delivers a smoother, more gradual sourness [18]. These acids influence the pH gradient of the fruit’s cell sap due to their distinct dissociation constants (pKa values of 3.4 for malic acid and 3.1 for citric acid). In this study, the organic acid content in both the pericarp and the pulp was analyzed. The results exhibited that malic acid accumulated rapidly in the early stages of fruit development, comprising over 95% of the organic acids at maturity, followed by citric acid. This high malic acid content is the primary contributor to the sour and astringent taste of *Cerasus humilis*. During the coloring–enlargement stage, the hard–ripe stage, and the fully ripe stage, the pericarp consistently had significantly higher levels of malic and citric acids than the pulp, indicating a distinct difference in acidity. The malic acid content peaked during the hard–ripe stage and then declined at maturity. Similarly, citric acid accumulated during the hard seed stage, peaked during the coloring–enlargement stage, and then gradually decreased [19]. This shift in organic acid content is likely linked to changes in metabolic pathways and the distribution of carbon sources. In the early stages of development, fruit cells actively divide and expand, using malic and citric acids as metabolic intermediates for synthesizing amino acids, lipids, and other cellular components. As the fruit matures, metabolic processes shift toward the pentose phosphate pathway (PPP) and gluconeogenesis, with sugars and lipids taking precedence, leading to a reduction in organic acids [20]. Additionally, the increase in fruit size and water content likely dilutes the concentration of organic acids, contributing further to the decrease in acidity [21].

### 3.2. Changes in Organic Acid Metabolism-Related Enzymes in the Pericarp and the Pulp of Cerasus humilis During Development

Organic acid metabolism in fruit development is highly dynamic, with fluctuations in malic and citric acid content largely driven by the coordinated action of several enzymes. Previous studies have indicated that enzymes such as phosphoenolpyruvate carboxylase (PEPC), malate dehydrogenase (NAD-MDH), NADP-malic enzyme (NADP-ME), citrate synthase (CS), and aconitase (ACO) are crucial in the synthesis and breakdown of malic acid and citric acid during fruit development [22,23]. To examine their regulatory roles, we measured the activity of these enzymes. PEPC activity in the pericarp of *Cerasus humilis* initially increased, then decreased, and later increased again during development. By contrast, enzyme activity in the pulp exhibited a rise followed by a decline. This suggests the differing regulatory effects of PEPC on organic acid metabolism in the pericarp and the pulp. Additionally, malic acid content in both parts was positively correlated with PEPC activity, with a strong correlation in the pericarp. However, citric acid content was either negatively correlated or not correlated, indicating that PEPC activity primarily influences malic acid accumulation without affecting citric acid accumulation. This aligns with previous findings [24].

Malate dehydrogenase (NAD-MDH) supports malic acid accumulation, while NADP-malic enzyme (NADP-ME) facilitates malic acid breakdown [25]. In this study, malic acid accumulation in both the pericarp and the pulp followed the same trend as NAD-MDH activity, showing an initial increase followed by a decrease, and was significantly positively correlated. This suggests that NAD-MDH promotes malic acid synthesis in both the pericarp and the pulp of *Cerasus humilis*. NADP-ME activity in the pericarp was negatively correlated with malic acid accumulation, indicating that it promotes malic acid breakdown. By contrast, NADP-ME activity in the pulp did not correlate with malic acid content, possibly due to the high activity of PEPC and NAD-MDH, leading to overproduction of malic acid and disturbing the balance between synthesis and degradation. NADP-ME activity in the pulp was significantly higher than in the pericarp during the transition from the hard seed stage to the fully ripe stage, promoting increased breakdown of malic acid in the pulp. This likely accounts for the higher acidity in the pericarp compared to the pulp. NADP-ME plays a critical role in the difference in malic acid content between the pericarp and the pulp.

Previous studies have suggested that CS and ACO play key roles in regulating citric acid levels, with CS promoting citric acid accumulation and ACO involved in its degradation [26]. In this study, CS activity in the pericarp of *Cerasus humilis* first increased and then decreased, while ACO activity initially decreased and then increased. The two enzyme activities were positively correlated and negatively correlated with citric acid content, respectively. This indicates that CS promotes citric acid accumulation, and ACO is a key enzyme regulating citric acid content in the pericarp. In the pulp, CS activity increased initially and then decreased, while ACO activity gradually increased, both negatively correlating with citric acid content. This pattern may be due to lower ACO activity at the young fruit stage, resulting in a higher proportion of citric acid. The higher citric acid concentration inhibits CS activity through feedback, a phenomenon also seen in passion fruit [22] and plums [27]. Throughout development, CS activity was significantly higher in the pericarp than in the pulp, while ACO activity exhibited little difference, explaining the higher citric acid content in the pericarp. CS is the key enzyme responsible for the difference in citric acid content between the pericarp and the pulp.

### 3.3. Acid Accumulation, Metabolic Enzymes, and Gene Expression Dynamics During Pericarp and Pulp Development in Cerasus humilis

Organic acid content, enzyme activity, and the expression of their encoding genes are dynamically correlated [15]. In this study, *ChPEPC* positively regulates PEPC activity in both the pericarp and the pulp. PEPC activity strongly correlates with malic acid content, especially in the pericarp (*p* < 0.01), and negatively correlates with citric acid content in both tissues. This suggests that *ChPEPC* enhances PEPC synthesis, promoting malic acid accumulation and playing a crucial role in regulating its differential metabolism in the pericarp and the pulp. Both the *ChMDH1* and *ChMDH2* genes positively regulate MDH activity in the pericarp and the pulp. MDH activity correlates positively with malic acid content, suggesting that the expression of these genes promotes the synthesis of MDH, regulating malic acid metabolism and its accumulation. The high expression levels of *ChMDH1* and *ChMDH2* in both tissues indicate that these genes are essential for malic acid synthesis in the pericarp and the pulp. In the pericarp, *ChME* expression is positively correlated with ME activity, which negatively correlates with malic acid content. In the pulp, however, *ChME* expression is inversely related to ME activity, which also negatively correlates with malic acid content. This indicates that the differential expression of *ChME* in the pericarp and the pulp affects enzyme activity and malic acid content, making *ChME* a key gene responsible for variations in malic acid levels between these two tissues. In the pericarp, *ChCS1* and *ChCS2* enhance CS activity, while *ChCS3* inhibits it. CS activity positively correlates with citric acid content. In the pulp, *ChCS1* and *ChCS3* promote CS activity, while *ChCS2* suppresses it. CS activity, in turn, negatively correlates with citric acid content. These findings suggest that the differential expression of *ChCS1*, *ChCS2*, and *ChCS3* between the pericarp and the pulp influences CS activity, which in turn affects citric acid accumulation in each tissue. *ChCS3* and *ChCS2* are key genes responsible for citric acid synthesis in the pericarp and the pulp, respectively, and contribute to the differential citric acid content between these two tissues. In both tissues, *ChACO1* enhances ACO activity, while *ChACO2* suppresses it. ACO activity negatively correlates with citric acid content, with a significant correlation observed in the pericarp (*p* < 0.05). These results suggest that *ChACO1* and *ChACO2* together regulate citric acid content by modulating ACO activity.

To sum up, *ChMDH1* and *ChMDH2* are essential for promoting malic acid synthesis in both the pericarp and the pulp, *ChME* is a differential gene responsible for variations in malic acid content, and *ChCS3* and *ChCS2* are key genes regulating citric acid synthesis in the pericarp and the pulp, respectively, contributing to the differential citric acid content between the two tissues. These findings provide a foundation for further research.

## 4. Materials and Methods

### 4.1. Experimental Site

In this experiment, the MY-23 cultivar of *Cerasus humilis* was used as the material. These materials were planted at the *Cerasus humilis* research base of Inner Mongolia Agricultural University, located in Hohhot, Inner Mongolia, at a longitude of 111.65° and a latitude of 40.82°. The region has a mid-temperate continental monsoon climate with four distinct seasons, an average annual temperature of about 6.5 °C, annual precipitation of about 400 mm, warm and humid summers with lots of rain, and cold and dry winters with a large temperature difference between day and night.

### 4.2. Sample Collection

From June to September 2023, the MY-23 cultivar of *Cerasus humilis* was used as the experimental material. Flowering time was recorded on 24 April. The fruit development stages include the young fruit stage (S1, 10 June), the hard seed stage (S2, 10 July), the coloring–enlargement stage (S3, 20 August), the hard–ripe stage (S4, 5 September), and the fully ripe stage (S5, 20 September). Harvested fruit are uniformly consistent in quality and size, and dark red in color. Disease-free, pest-free, and mechanically undamaged fruits were selected for study. The fruits were washed with distilled water, air-dried, and then evenly divided into three parts. The fruits were separated into pericarp and pulp, then quickly frozen with liquid nitrogen and stored at −80 °C for further analysis.

### 4.3. Experimental Index Measurement

#### 4.3.1. Determination of Organic Acids

Organic acid content was determined using the method described by Nour [28], with modifications.

The organic acid content of the pulp was measured as follows: 3.0 g of pulp, thoroughly ground and homogenized with 3 mL of ultrapure water, was transferred to a 10 mL centrifuge tube. The mixture was heated in a water bath at 80 °C for 45 min for water bath extraction and then centrifuged at 12,000 rpm for 10 min at 4 °C. The supernatant was collected. Then, 2 mL of ultrapure water was added to the residue, mixed thoroughly, and extracted again under the same conditions for 45 min. After centrifugation, the two supernatants were combined, adjusted to a volume of 5 mL, and filtered through a 0.45 µm membrane. The extract was analyzed by UPLC.

The organic acid content of the pericarp was measured as follows: 0.5 g of pericarp, thoroughly ground and homogenized with 2 mL of ultrapure water, was transferred to a 10 mL centrifuge tube. The mixture was heated in a water bath at 80 °C for 45 min for water bath extraction and then centrifuged at 12,000 rpm for 10 min at 4 °C. The supernatant was collected. Then, 2 mL of ultrapure water was added to the residue, mixed thoroughly, and extracted again under the same conditions for 45 min. After centrifugation, the above procedure was repeated again and the three supernatants were combined, adjusted to a volume of 6 mL and filtered through a 0.45 µm membrane. The extract was analyzed by UPLC.

HPLC conditions: Chromatographic separation was carried out on an Ultimate LP-C18 column (4.6 × 300 mm, 5 µm). The mobile phase consisted of 0.01 mol/L potassium dihydrogen phosphate aqueous solution and methanol (95: 5, *v*/*v*). The column temperature was maintained at 30 °C, with a flow rate of 0.5 mL/min. A 10 µL aliquot was injected for each run, and detection was performed at a wavelength of 210 nm. The total run time for each analysis was 30 min.

#### 4.3.2. Measurement of the Activity of Acid Metabolism-Related Enzymes

The activities of NADP-ME, NAD-MDH, CS, PEPC, and ACO in the pericarp and the pulp of *Cerasus humilis* were measured using assay kits for malate dehydrogenase (NADP-ME, BC1120-50T/48S), malate dehydrogenase (NAD-MDH, BC1055-100T/96S), phosphoenolpyruvate carboxylase (PEPC, BC3315-100T/96S), citrate synthase (CS, BC1065-100T/48S), and aconitase (Mit-ACO, BC4485-100T/96S) (Solarbio Biochemical Assay Kits, Beijing, China, https://www.solarbio.com/).

### 4.4. Correlation Analysis Methods

Using Pearson correlation analysis, the Pearson correlation coefficient takes values between [-1 and 1]; when r > 0, it indicates that there is a positive correlation between the two variables; when r < 0, there is a negative correlation between the two variables. Significant correlation was defined as |rho| ≥ 0.5 and *p* < 0.05. The data matrix was constructed based on the experimental part and the observation phase, and all variables were included in a single matrix for analysis.

### 4.5. Screening of Genes Related to Organic Acid Metabolism Was Conducted Using Transcriptome Sequencing

#### 4.5.1. Experimental Materials

The pericarp of the fruit at the coloring–enlargement stage is abbreviated as S3GP, the pulp of the fruit at the coloring–enlargement stage is abbreviated as S3GR; the pericarp of the fruit at the hard–ripe stage is abbreviated as S4GP, and the pulp of the fruit at the hard–ripe stage is abbreviated as S4GR.

#### 4.5.2. RNA Extraction

The plant total RNA was extracted using the RNAprep Pure Plant Kit (Tiangen, Beijing, China) according the instructions provided by the manufacturer.

#### 4.5.3. Library Construction

After the samples were qualified, library construction was carried out, and the main processes were as follows: enrich eukaryotic mRNA with magnetic beads with Oligo (dT); add fragmentation buffer to interrupt the mRNA randomly; use mRNA as a template, synthesize the first cDNA strand and the second strand, and carry out cDNA purification; purified double-stranded cDNA was then subjected to end repair, addition of A tail, and ligation of sequencing junctions; then, use AMPure XP beads for fragment size selection; and then, use AMPure XP beads to select the fragment size. The purified double-stranded cDNA was then subjected to end repair, addition of A-tail, and ligation of sequencing junctions, followed by fragment size selection with AMPure XP beads; finally, the cDNA library was enriched by PCR.

#### 4.5.4. RNA Quantification and Qualification

RNA concentration and purity was measured using NanoDrop™ 2000 (Thermo Fisher Scientific, Wilmington, DE, USA). RNA integrity was assessed using the RNA Nano 6000 Assay Kit of the Agilent Bioanalyzer 2100 system (Agilent Technologies, Santa Clara, CA, USA).

#### 4.5.5. Library Preparation for Transcriptome Sequencing

A total amount of 1 μg RNA per sample was used as input material for the RNA sample preparations. Sequencing libraries were generated using Hieff NGS™ Ultima Dual-mode mRNA Library Prep Kit for Illumina (Yeasen Biotechnology (Shanghai) Co., Ltd., Shanghai, China), following the manufacturer’s recommendations, and index codes were added to attribute sequences to each sample. Briefly, mRNA was purified from total RNA using poly-T oligo-attached magnetic beads. First strand cDNA was synthesized and second strand cDNA synthesis was subsequently performed. Remaining overhangs were converted into blunt ends via exonuclease/polymerase activities. After adenylation of 3′ ends of DNA fragments, NEBNext Adaptor with hairpin loop structure were ligated to prepare for hybridization. The library fragments were purified with AMPure XP system (Beckman Coulter, Beverly, CA, USA). Then 3 μL USER Enzyme (NEB, Ipswich, MA, USA) was used with size-selected, adaptor-ligated cDNA at 37 °C for 15 min followed by 5 min at 95 °C before PCR. Then PCR was performed with Phusion High-Fidelity DNA polymerase, Universal PCR primers and Index (X) Primer. At last, PCR products were purified (AMPure XP system), and library quality was assessed on the Agilent Bioanalyzer 2100 system.

#### 4.5.6. Sequencing

The library was sequenced on the Illumina NovaSeq platform, generating 150 bp paired-end reads. The transcriptome sequencing was completed by Beijing Biomarker Technologies Co., Ltd., Beijing, China.

##### Quality Control

Raw data (raw reads) of fastq format were firstly processed through in-house perl scripts. In this step, clean data (clean reads) were obtained by removing reads containing adapter, reads containing ploy-N, and low quality reads from raw data. At the same time, Q20, Q30, GC-content, and sequence duplication level of the clean data were calculated. All the downstream analyses were based on clean data with high quality.

##### Reads Mapping to the Reference Genome

This project used the specified genome as a reference for sequence alignment and subsequent analysis. The version information of the reference genome is the *Prunus_humilis*.v1.genome.fa.

Baimaike used the HISAT2 [29] software to rapidly and accurately align the clean reads to the reference genome, obtaining the positional information of the reads on the reference genome. Then, StringTie [30] was used to assemble the aligned reads and reconstruct the transcriptome for subsequent analysis.

##### Novel Transcripts Prediction and Gene Functional Annotation

The StringTie Reference Annotation-Based Transcript (RABT) assembly method was used to construct and identify both known and novel transcripts from Hisat2 alignment results.

Gene function was annotated based on the following databases: Nr (NCBI non-redundant protein sequences); Pfam (Protein family); KOG/COG (Clusters of Orthologous Groups of proteins); Swiss-Prot (A manually annotated and reviewed protein sequence database); KO (KEGG Ortholog database); GO (Gene Ontology).

##### Quantification of Gene Expression Levels

The number of mapped reads and transcript length in the samples were normalized. Here, we used StringTie with the maximum flow algorithm and employed FPKM (fragments per kilobase of transcript per million fragments mapped) for normalization, which serves as an indicator for measuring the expression level of transcripts or genes.

(1) $$\mathrm{FPKM}=\frac{\mathrm{cDNA}\;\mathrm{Fragments}}{\mathrm{Mapped}\;\mathrm{Fragments}\left(\mathrm{Millions}\right)\times \mathrm{TranscriptLength}\left(\mathrm{kb}\right)}$$

##### Differential Expression Analysis

Differential expression analysis of two conditions/groups was performed using the DESeq2. DESeq2 provides statistical routines for determining differential expression in digital gene expression data using a model based on the negative binomial distribution. The resulting *p*-values were adjusted using the Benjamini–Hochberg approach for controlling the false discovery rate.

Genes with an adjusted *p*-value < 0.01 and fold change ≥ 2 found by DESeq2 were assigned as differentially expressed.

##### GO Enrichment Analysis

Gene Ontology (GO) enrichment analysis of the differentially expressed genes (DEGs) was implemented by the clusterProfiler packages based Wallenius non-central hyper-geometric distribution, which can adjust for gene length bias in DEGs.

##### KEGG Pathway Enrichment Analysis

KEGG (Kyoto Encyclopedia of Genes and Genomes) is a resource that helps in understanding the high-level functions and utilities of biological systems, such as cells, organisms, and ecosystems, from molecular-level information, particularly large-scale molecular datasets generated by genome sequencing and other high-throughput experimental techniques (http://www.genome.jp/kegg/, accessed on 23 January 2025). In this study, we used the KOBAS database and the clusterProfiler (4. 14. 6) software to test the statistical enrichment of differentially expressed genes in KEGG pathways.

#### 4.5.7. RT-qPCR Validation

For RT-qPCR validation, screened genes related to organic acid metabolism were selected and primers were created for specific genes using the Actin1 gene as an internal standard using the NCBI website, the primers were synthesized Sangon Biotech (Sangon Biotech (Shanghai) Co., Ltd., Shanghai, China) (Table 3). RT-qPCR reactions were performed using the 2×HQ SYBR qPCR Mix (Low ROX), (ZF502, Beijing Zoman Biotechnology Co., Ltd., Beijing, China) and the Q2000C Real-Time qPCR System (Hangzhou LongGene Scientific Instruments Co., Ltd., Hangzhou, China).

#### 4.5.8. Data Analysis

Data analysis was performed using Excel and IBM SPSS Statistics 27, and graphs were generated using GraphPad Prism 8.

The raw reads were processed further using the bioinformatics pipeline tool on the BMKCloud online platform (www.biocloud.net).

## 5. Conclusions

Malic acid is the predominant organic acid, with citric acid being the secondary one, in both the pericarp and the pulp of *Cerasus humilis*. The difference in acidity between the two is jointly determined by the malic acid and citric acid content. MAD-MDH and CS are the key enzymes involved in the synthesis of malic acid and citric acid in the fruit pericarp and the pulp. The activity levels of NADP-ME and CS are the primary factors determining the differences in malic acid and citric acid content in the pericarp and the pulp. *ChMDH1* and *ChMDH2* are the key genes promoting malic acid synthesis in both the pericarp and the pulp, while *ChME* is the differential gene responsible for the variation in malic acid content between the pericarp and the pulp. *ChCS3* and *ChCS2* are the key genes for citric acid synthesis in the pericarp and the pulp, respectively, and are also the differential genes responsible for the differences in citric acid content.

## Figures and Tables

**Table 1:** Statistical table of functional annotation counts for differentially expressed genes in each database.

DEG Set	Total	COG	GO	KEGG	KOG	NR	Pfam	Swiss-Prot	eggNOG
S3GP_vs_S3GR	5838	1871	4877	3906	3116	5833	4359	4143	4958
S4GP_vs_S4GR	7051	2162	5925	4778	3936	7043	5283	4979	6021

Note: S3: the coloring–enlargement stage; S4: the hard–ripe stage; GP: the pericarp of *Cerasus humilis*; GR: the pulp of *Cerasus humilis*.

**Table 2:** List of gene prediction names.

Numbering of New Genes	Predicting Enzyme-Encoding Genes	Genes Name
NewGene_1191.1	Malate dehydrogenase gene	*ChMDH1*
NewGene_6957.1	Malate dehydrogenase gene	*ChMDH2*
NewGene_20719.2	Malic enzyme gene	*ChME*
NewGene_10906.1	Phosphoenolpyruvate carboxykinase gene	*ChPEPC*
NewGene_3681.1	Aconitic acid hydratase gene	*ChACO1*
NewGene_13406.1	Aconitic acid hydratase gene	*ChACO2*
NewGene_14300.1	Citrate synthase gene	*ChCS1*
NewGene_18025.5	Citrate synthase gene	*ChCS2*
NewGene_14300.1	Citrate synthase gene	*ChCS3*

**Table 3:** List of primers.

Numbering of New Genes	Forward Primer	Reverse Primer
NewGene_1191.1 (*ChMDH1*)	gcg gtg ctg caa tca tca agg	aga agg aac att gta cga gcc atc a
NewGene_6957.1 (*ChMDH2*)	gaa gtt gga tgc aac cgc ag	aac tta gaa cca cgc cca gg
NewGene_20719.2 (*ChME*)	agg cga aga cca ttg tca ag	tca gaa atg gcg gca aaa gc
NewGene_10906.1 (*ChPEPC*)	gta aac gca gtg cag gca aa	cca gga gga acg aca agc at
NewGene_3681.1 (*ChACO1*)	atc aca act gac cac atc tca cct g	atc tca tca cca cgg cga ctt c
NewGene_13406.1 (*ChACO2*)	gta aac gca gtg cag gca aa	cca gga gga acg aca agc at
NewGene_14300.1 (*ChCS1*)	gct gct ctg aag caa cca ac	ctc gtg caa acg cga tca at
NewGene_18025.5 (*ChCS2*)	gag gtg gag atg ggt ggt tg	atg ctg ttc cca agt cgg tc
NewGene_14300.1 (*ChCS3*)	gga cta tgg tag ctg gag gag gt	gca caa tca atc aca act ctg gca t
Actin1	gca gcg act gaa gac ata caa g	ggt ggc att agc aag ttc ctt

## Data Availability

The data are contained within this article.
